# The New Health Physician

**Published:** 1888-02

**Authors:** 


### Editorial.

### THE NEW HEALTH PHYSICIAN.

The wheel of politics has made another revolution, resulting in the removal from office of one of the ablest and most enthusiastic health physicians it has ever been the fortune of our city to possess.

When, four years ago, Dr. A. H. Briggs assumed charge of the sanitary affairs of Buffalo, there was practically no Health Department in existence. Vital statistics were scarcely recorded, there were no health inspectors to search out and report on public nuisances, while the death-rate for the two years preceding his appointment averaged over twenty-three per 1,000. Dr. Briggs reorganized the department, so that no city in the Union has more accurate vital statistics; a thorough system of inspection was inaugurated, which has resulted in preventing epidemics of small-pox, a disease which has three times during his term of office been brought into the city, but in no instance did a single person outside of the family where the disease appeared contract the disease. The death-rate during the past four years has been only a little over nineteen per 1,000.

The citizens are, however, to be congratulated that an able and intelligent successor has been appointed. Dr. Edward Clark is one of the brightest and most active of the younger physicians of Buffalo, and will, undoubtedly, be able to fully maintain the excellent condition of the Health Department, which his predecessor succeeded in establishing. Dr. Clark has already instituted some measures which will result in improving the sanitary condition of the public schools, a reform which will be of incalculable benefit to our citizens. Being intimately acquainted with our health physicians, past and present, our sentiments toward them are very much like those of French in former times, when they had exchanged kings: "*Le Roi est mort. Vive le Roi!*"

